# SISEA activities in Pasteur Institute in Nha Trang, Vietnam, during 2008–2009

**Published:** 2011-01-10

**Authors:** Bui Trong Chien, Vien Quang Mai, Trinh Thi Xuan Mai, Nguyen Hai Nam, Ðoan Thi thanh Thuy

**Affiliations:** 1 Pasteur Institute of Nha Trang, Vietnam

## Background

In recent years, the situation of dangerous infectious disease has developed more complex. Some emerging infectious diseases such as Severe Acute Respiratory Syndrome (SARS), avian influenza tend to globalization. In Central Vietnam, a lot of infectious diseases circulating every year caused serious consequences such as SARI, VE, DF/DHF, etc. For SARI, except influenza virus which has monitored, the other respiratory pathogens have not been concerned yet. It’s difficult to detect some agents cause disease in regional and provincial laboratory. In order to respond the complex development of emerging diseases such as SARS, avian influenza, severe acute respiratory infection (SARI), viral encephalitis (VE) and Dengue fever/Dengue haemorrhagic fever, Pasteur Institute Network in Southeast Asia has implemented the project “Surveillance and Investigation of epidemic situation in South East Asia (SISEA)”.

## Results

A specific surveillance system for Severe Acute Respiratory Infection (SARI) was established in 02 sentinel hospitals in Bin Dinh province from January 2008 to December 2009. Patient’s signature on written ICF had been required before the enrollment and samples (nasopharyngeal swabs) were analyzed by RT-PCR multiplex. There were 1,155 cases of SARI enrolled, with 47.2% testing positive for one of the 17 respiratory viruses included in our panel. Rhinvoirus (24%) and respiratory syncitial virus (18%) accounted for almost 50% of the positive samples. Adenovirus, human metapneumovirus and NL63 coronavirus were present in 5-6% of the samples, whereas other respiratory viruses showed lower incidence. Of note, only few samples tested positive for pandemic H1N1 influenza. In addition, we carried out a number of training and supervision sessions for the personnel of the sentinel hospitals, both in field epidemiology and molecular laboratory techniques.

## Conclusion

This project is strengthening laboratory capability as well as epidemiological surveillance system to enable rapid diagnosis and prevention of dangerous epidemics, thus helping to contain their spread in the region. This is really necessary and responds to current practical needs in Central Vietnam.

